# 3-(*tert*-But­oxy­carbon­yl)-2-(4-chloro­phen­yl)-1,3-thia­zolidine-4-carb­oxy­lic acid

**DOI:** 10.1107/S1600536810037396

**Published:** 2010-09-25

**Authors:** Shu-Min Ding

**Affiliations:** a School of Chemical Engineering, Changzhou University, Changzhou 213164, People’s Republic of China

## Abstract

In the title compound, C_15_H_18_ClNO_4_S, the thia­zolidine ring adopts a twisted conformation about the S—C(methyl­ene) bond. The dihedral angle between the five- and six-membered rings is 77.2 (3)°. In the crystal, the mol­ecules are linked by O—H⋯O hydrogen bonds, generating *C*(7) chains propagating in [100].

## Related literature

For background to the biological properties of the title compound, see: Lu *et al.* (2010 ▶); Song *et al.* (2009 ▶). For reference bond-length data, see: Allen *et al.* (1987 ▶). 
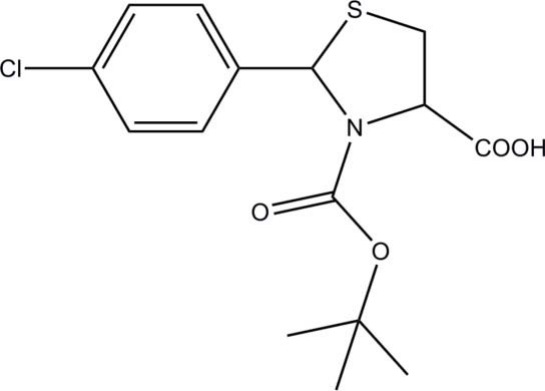


## Experimental

### 

#### Crystal data

C_15_H_18_ClNO_4_S
*M* *_r_* = 343.81
Monoclinic, 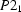

*a* = 6.4600 (13) Å
*b* = 10.641 (2) Å
*c* = 12.411 (3) Å
β = 94.52 (3)°
*V* = 850.5 (3) Å^3^
*Z* = 2
Mo *K*α radiation
μ = 0.36 mm^−1^
*T* = 293 K
0.30 × 0.20 × 0.10 mm

#### Data collection

Enraf–Nonius CAD-4 diffractometer
Absorption correction: ψ scan (North *et al.*, 1968 ▶) *T*_min_ = 0.899, *T*_max_ = 0.965
1638 measured reflections
1638 independent reflections
1363 reflections with *I* > 2σ(*I*)
200 standard reflections every 3 reflections  intensity decay: 1%

#### Refinement

*R*[*F* ^2^ > 2σ(*F* ^2^)] = 0.062
*wR*(*F* ^2^) = 0.159
*S* = 1.08
1638 reflections
185 parameters
89 restraints
H-atom parameters constrained
Δρ_max_ = 0.43 e Å^−3^
Δρ_min_ = −0.37 e Å^−3^
Absolute structure: Flack (1983 ▶)
Flack parameter: −0.09 (19)

### 

Data collection: *CAD-4 Software* (Enraf–Nonius, 1989 ▶); cell refinement: *CAD-4 Software*; data reduction: *XCAD4* (Harms & Wocadlo, 1995 ▶); program(s) used to solve structure: *SHELXS97* (Sheldrick, 2008 ▶); program(s) used to refine structure: *SHELXL97* (Sheldrick, 2008 ▶); molecular graphics: *SHELXTL* (Sheldrick, 2008 ▶); software used to prepare material for publication: *SHELXTL*.

## Supplementary Material

Crystal structure: contains datablocks global, I. DOI: 10.1107/S1600536810037396/hb5637sup1.cif

Structure factors: contains datablocks I. DOI: 10.1107/S1600536810037396/hb5637Isup2.hkl

Additional supplementary materials:  crystallographic information; 3D view; checkCIF report

## Figures and Tables

**Table 1:** Hydrogen-bond geometry (Å, °)

*D*—H⋯*A*	*D*—H	H⋯*A*	*D*⋯*A*	*D*—H⋯*A*
O2—H2*A*⋯O3^i^	0.82	1.83	2.638 (6)	167

Symmetry code: (i) 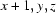.

**Fig. 1.**
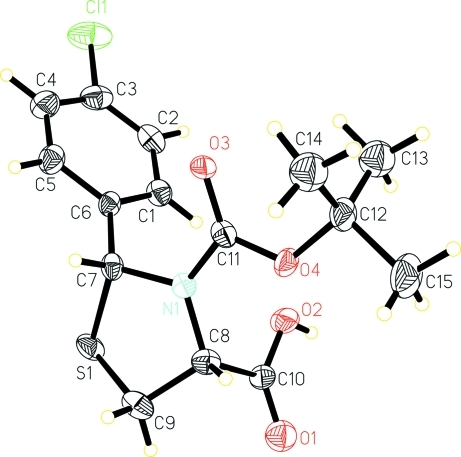
The structure of (I) showing 30% probability displacement ellipsoids.

## supplementary crystallographic information

### Comment

Recently, 3-*tert*-butoxycarbonyl-2-arylthiazolidine-4-carboxylic acid derivatives have been reported to possess antimicrobial and antitumor activities (Song *et al.*, 2009; Lu *et al.*, 2010). In this work, we report here the crystal structure of the title compound, (I). In (I), all bond lengths are within normal ranges (Allen *et al.*, 1987) (Fig. 1). There are intermolecular O—H···O hydrogen bonds in (I).

### Experimental

A mixture of *L*-cysteine (1.41 g, 10 mmol) and 4-chlorobenzaldehyde (1.4 g, 10 mmol) in methanol (100 ml) was stirred at room temperature for 10 h, and the separated solid was collected, washed with diethyl ether, and dried to obtain 2-(4-chlorophenyl)thiazolidine-4-carboxylic with yield of 90%. In ice water, 2-(4-chlorophenyl)thiazolidine-4-carboxylic (1 mmol) was dissolved in 1 N NaOH (1 ml) and 1,4-dioxane (10 ml); then di-*tert*-butyldicarbonate (1 mmol) was added slowly and stirred at room temperature for 6 h. The reaction mixture was concentrated in a vacuum and washed with ethyl acetate (10 ml). The aqueous phase was adjusted to pH 4 by adding 1 N HCl, then extracted with ethyl acetate, dried with magnesium sulfate, filtered, After keeping the filtrate in air for 5 d, colorless block-shaped crystals of (I) were formed.

### Refinement

All H atoms were positioned geometrically (C—H = 0.93 Å for the aromatic H atoms and C—H = 0.96 Å for the aliphatic H atoms) and were refined as riding, with *U*_iso_(H) = 1.2*U*_eq_(C) and *U*_iso_(H) = 1.2*U*_eq_(N).

### Crystal data

**Table:**

C_15_H_18_ClNO_4_S	*F*(000) = 360
*M**_r_* = 343.81	*D*_x_ = 1.343 Mg m^−^^3^
Monoclinic, *P*2_1_	Mo *K*α radiation, λ = 0.71073 Å
Hall symbol: P 2yb	Cell parameters from 25 reflections
*a* = 6.4600 (13) Å	θ = 9–12°
*b* = 10.641 (2) Å	µ = 0.36 mm^−^^1^
*c* = 12.411 (3) Å	*T* = 293 K
β = 94.52 (3)°	Block, colorless
*V* = 850.5 (3) Å^3^	0.30 × 0.20 × 0.10 mm
*Z* = 2	

### Data collection

**Table:**

Enraf–Nonius CAD-4 diffractometer	1363 reflections with *I* > 2σ(*I*)
Radiation source: fine-focus sealed tube	*R*_int_ = 0.0000
graphite	θ_max_ = 25.3°, θ_min_ = 1.7°
ω/2θ scan	*h* = −7→7
Absorption correction: ψ scan (North *et al.*, 1968)	*k* = 0→12
*T*_min_ = 0.899, *T*_max_ = 0.965	*l* = 0→14
1638 measured reflections	200 standard reflections every 3 reflections
1638 independent reflections	intensity decay: 1%

### Refinement

**Table:**

Refinement on *F*^2^	Secondary atom site location: difference Fourier map
Least-squares matrix: full	Hydrogen site location: inferred from neighbouring sites
*R*[*F*^2^ > 2σ(*F*^2^)] = 0.062	H-atom parameters constrained
*wR*(*F*^2^) = 0.159	*w* = 1/[σ^2^(*F*_o_^2^) + (0.0649*P*)^2^ + 1.2912*P*] where *P* = (*F*_o_^2^ + 2*F*_c_^2^)/3
*S* = 1.08	(Δ/σ)_max_ < 0.001
1638 reflections	Δρ_max_ = 0.43 e Å^−^^3^
185 parameters	Δρ_min_ = −0.37 e Å^−^^3^
89 restraints	Absolute structure: Flack (1983)
Primary atom site location: structure-invariant direct methods	Flack parameter: −0.09 (19)

### Special details

**Table:**

Geometry. All e.s.d.'s (except the e.s.d. in the dihedral angle between two l.s. planes) are estimated using the full covariance matrix. The cell e.s.d.'s are taken into account individually in the estimation of e.s.d.'s in distances, angles and torsion angles; correlations between e.s.d.'s in cell parameters are only used when they are defined by crystal symmetry. An approximate (isotropic) treatment of cell e.s.d.'s is used for estimating e.s.d.'s involving l.s. planes.
Refinement. Refinement of *F*^2^ against ALL reflections. The weighted *R*-factor *wR* and goodness of fit *S* are based on *F*^2^, conventional *R*-factors *R* are based on *F*, with *F* set to zero for negative *F*^2^. The threshold expression of *F*^2^ > σ(*F*^2^) is used only for calculating *R*-factors(gt) *etc*. and is not relevant to the choice of reflections for refinement. *R*-factors based on *F*^2^ are statistically about twice as large as those based on *F*, and *R*- factors based on ALL data will be even larger.

### Fractional atomic coordinates and isotropic or equivalent isotropic displacement parameters (Å^2^)

**Table:**

	*x*	*y*	*z*	*U*_iso_*/*U*_eq_	
C1	0.3575 (11)	0.4607 (7)	0.7980 (6)	0.0457 (16)	
H1	0.4776	0.4984	0.7768	0.055*	
C2	0.3229 (12)	0.3336 (8)	0.7816 (6)	0.0519 (17)	
H2	0.4183	0.2863	0.7465	0.062*	
C3	0.1505 (12)	0.2766 (8)	0.8162 (7)	0.0555 (18)	
C4	0.0021 (13)	0.3440 (8)	0.8654 (6)	0.0585 (18)	
H4	−0.1163	0.3054	0.8879	0.070*	
C5	0.0368 (11)	0.4720 (7)	0.8800 (6)	0.0492 (16)	
H5	−0.0604	0.5195	0.9136	0.059*	
C6	0.2109 (9)	0.5308 (6)	0.8464 (5)	0.0349 (13)	
C7	0.2361 (9)	0.6677 (6)	0.8692 (4)	0.0358 (13)	
H7	0.1036	0.7023	0.8887	0.043*	
C8	0.4770 (9)	0.8351 (7)	0.8161 (5)	0.0409 (14)	
H8	0.4364	0.9165	0.7842	0.049*	
O1	0.8408 (8)	0.8639 (5)	0.8109 (4)	0.060	
C9	0.4827 (11)	0.8471 (7)	0.9375 (5)	0.0494 (16)	
H9A	0.3760	0.9043	0.9583	0.059*	
H9B	0.6170	0.8774	0.9671	0.059*	
C10	0.6910 (9)	0.8031 (8)	0.7835 (5)	0.0459 (17)	
C11	0.2163 (9)	0.7473 (7)	0.6838 (5)	0.0373 (14)	
C12	0.2095 (11)	0.8500 (8)	0.5098 (5)	0.0520 (18)	
C13	0.2564 (14)	0.7386 (9)	0.4443 (8)	0.070	
H13A	0.3999	0.7161	0.4586	0.106*	
H13B	0.2293	0.7579	0.3690	0.106*	
H13C	0.1705	0.6695	0.4629	0.106*	
C14	−0.0195 (13)	0.8803 (9)	0.4998 (8)	0.069	
H14A	−0.0975	0.8056	0.5123	0.104*	
H14B	−0.0583	0.9117	0.4286	0.104*	
H14C	−0.0483	0.9429	0.5523	0.104*	
C15	0.3316 (15)	0.9649 (10)	0.4750 (7)	0.078 (3)	
H15A	0.2943	1.0371	0.5156	0.116*	
H15B	0.2992	0.9802	0.3993	0.116*	
H15C	0.4777	0.9492	0.4883	0.116*	
Cl1	0.1156 (5)	0.1156 (2)	0.7961 (2)	0.0908 (9)	
N1	0.3167 (7)	0.7456 (5)	0.7848 (4)	0.0336 (11)	
O2	0.6980 (6)	0.7001 (6)	0.7256 (4)	0.0534 (12)	
H2A	0.8185	0.6858	0.7131	0.080*	
O3	0.0721 (6)	0.6749 (5)	0.6573 (3)	0.0398 (10)	
O4	0.2916 (7)	0.8324 (5)	0.6216 (3)	0.0448 (11)	
S1	0.4357 (3)	0.68989 (18)	0.98363 (12)	0.0481 (5)	

### Atomic displacement parameters (Å^2^)

**Table:**

	*U*^11^	*U*^22^	*U*^33^	*U*^12^	*U*^13^	*U*^23^
C1	0.047 (4)	0.049 (3)	0.041 (4)	0.001 (3)	0.003 (3)	0.001 (3)
C2	0.059 (4)	0.055 (4)	0.041 (4)	0.010 (3)	0.001 (3)	−0.006 (3)
C3	0.064 (4)	0.047 (4)	0.053 (4)	−0.006 (3)	−0.014 (3)	0.007 (3)
C4	0.060 (4)	0.057 (4)	0.058 (4)	−0.014 (3)	0.004 (3)	0.004 (4)
C5	0.046 (3)	0.056 (4)	0.045 (4)	−0.004 (3)	0.003 (3)	0.011 (3)
C6	0.039 (3)	0.035 (3)	0.031 (3)	0.000 (2)	0.002 (2)	0.008 (3)
C7	0.035 (3)	0.043 (4)	0.030 (3)	0.005 (3)	0.003 (2)	0.006 (3)
C8	0.042 (3)	0.038 (3)	0.042 (3)	0.001 (3)	−0.004 (3)	0.001 (3)
O1	0.060	0.060	0.060	0.000	0.005	0.000
C9	0.052 (4)	0.052 (4)	0.043 (3)	0.008 (3)	−0.006 (3)	−0.009 (3)
C10	0.026 (3)	0.074 (5)	0.037 (3)	−0.015 (3)	−0.006 (2)	−0.002 (3)
C11	0.028 (3)	0.053 (4)	0.031 (3)	0.009 (3)	−0.002 (2)	−0.002 (3)
C12	0.050 (4)	0.070 (5)	0.035 (3)	−0.008 (4)	−0.003 (3)	0.019 (4)
C13	0.070	0.070	0.070	0.000	0.006	0.000
C14	0.070	0.070	0.070	0.000	0.006	0.000
C15	0.086 (6)	0.085 (7)	0.061 (5)	−0.007 (5)	0.006 (4)	0.036 (5)
Cl1	0.115 (2)	0.0475 (12)	0.1032 (19)	−0.0122 (13)	−0.0338 (16)	0.0021 (13)
N1	0.030 (2)	0.038 (3)	0.033 (2)	0.004 (2)	0.0010 (19)	0.001 (2)
O2	0.032 (2)	0.066 (3)	0.064 (3)	−0.010 (2)	0.012 (2)	−0.018 (3)
O3	0.029 (2)	0.058 (3)	0.0317 (19)	−0.007 (2)	−0.0026 (15)	−0.005 (2)
O4	0.044 (2)	0.055 (3)	0.035 (2)	−0.015 (2)	0.0001 (18)	0.016 (2)
S1	0.0578 (10)	0.0564 (10)	0.0285 (7)	−0.0005 (9)	−0.0071 (6)	−0.0025 (9)

### Geometric parameters (Å, °)

**Table:**

C1—C6	1.379 (9)	C9—H9A	0.9700
C1—C2	1.384 (11)	C9—H9B	0.9700
C1—H1	0.9300	C10—O2	1.313 (9)
C2—C3	1.367 (11)	C11—O3	1.234 (8)
C2—H2	0.9300	C11—O4	1.308 (8)
C3—C4	1.378 (12)	C11—N1	1.365 (8)
C3—Cl1	1.744 (8)	C12—O4	1.457 (8)
C4—C5	1.390 (11)	C12—C13	1.483 (12)
C4—H4	0.9300	C12—C14	1.510 (11)
C5—C6	1.380 (9)	C12—C15	1.535 (11)
C5—H5	0.9300	C13—H13A	0.9600
C6—C7	1.490 (9)	C13—H13B	0.9600
C7—N1	1.464 (8)	C13—H13C	0.9600
C7—S1	1.857 (6)	C14—H14A	0.9600
C7—H7	0.9800	C14—H14B	0.9600
C8—N1	1.437 (8)	C14—H14C	0.9600
C8—C10	1.510 (9)	C15—H15A	0.9600
C8—C9	1.510 (9)	C15—H15B	0.9600
C8—H8	0.9800	C15—H15C	0.9600
O1—C10	1.191 (8)	O2—H2A	0.8200
C9—S1	1.801 (8)		
			
C6—C1—C2	119.0 (7)	O1—C10—O2	123.2 (6)
C6—C1—H1	120.5	O1—C10—C8	122.8 (7)
C2—C1—H1	120.5	O2—C10—C8	114.0 (5)
C3—C2—C1	120.9 (7)	O3—C11—O4	125.6 (5)
C3—C2—H2	119.6	O3—C11—N1	122.1 (6)
C1—C2—H2	119.6	O4—C11—N1	112.3 (5)
C2—C3—C4	121.4 (8)	O4—C12—C13	110.1 (7)
C2—C3—Cl1	119.4 (7)	O4—C12—C14	112.7 (6)
C4—C3—Cl1	119.2 (7)	C13—C12—C14	111.5 (7)
C3—C4—C5	117.2 (8)	O4—C12—C15	102.4 (6)
C3—C4—H4	121.4	C13—C12—C15	110.5 (6)
C5—C4—H4	121.4	C14—C12—C15	109.2 (7)
C6—C5—C4	122.2 (8)	C12—C13—H13A	109.5
C6—C5—H5	118.9	C12—C13—H13B	109.5
C4—C5—H5	118.9	H13A—C13—H13B	109.5
C1—C6—C5	119.3 (6)	C12—C13—H13C	109.5
C1—C6—C7	122.9 (6)	H13A—C13—H13C	109.5
C5—C6—C7	117.8 (6)	H13B—C13—H13C	109.5
N1—C7—C6	117.2 (5)	C12—C14—H14A	109.5
N1—C7—S1	102.2 (4)	C12—C14—H14B	109.5
C6—C7—S1	109.2 (4)	H14A—C14—H14B	109.5
N1—C7—H7	109.3	C12—C14—H14C	109.5
C6—C7—H7	109.3	H14A—C14—H14C	109.5
S1—C7—H7	109.3	H14B—C14—H14C	109.5
N1—C8—C10	115.7 (6)	C12—C15—H15A	109.5
N1—C8—C9	106.7 (5)	C12—C15—H15B	109.5
C10—C8—C9	109.6 (5)	H15A—C15—H15B	109.5
N1—C8—H8	108.2	C12—C15—H15C	109.5
C10—C8—H8	108.2	H15A—C15—H15C	109.5
C9—C8—H8	108.2	H15B—C15—H15C	109.5
C8—C9—S1	104.3 (5)	C11—N1—C8	121.3 (5)
C8—C9—H9A	110.9	C11—N1—C7	119.6 (5)
S1—C9—H9A	110.9	C8—N1—C7	118.1 (5)
C8—C9—H9B	110.9	C10—O2—H2A	109.5
S1—C9—H9B	110.9	C11—O4—C12	121.8 (5)
H9A—C9—H9B	108.9	C9—S1—C7	90.0 (3)
			
C6—C1—C2—C3	−2.5 (11)	O3—C11—N1—C8	−176.7 (6)
C1—C2—C3—C4	2.1 (12)	O4—C11—N1—C8	3.6 (8)
C1—C2—C3—Cl1	−178.8 (6)	O3—C11—N1—C7	−8.1 (9)
C2—C3—C4—C5	−1.1 (11)	O4—C11—N1—C7	172.2 (5)
Cl1—C3—C4—C5	179.8 (6)	C10—C8—N1—C11	−83.3 (7)
C3—C4—C5—C6	0.5 (11)	C9—C8—N1—C11	154.5 (5)
C2—C1—C6—C5	1.9 (10)	C10—C8—N1—C7	107.9 (6)
C2—C1—C6—C7	178.9 (6)	C9—C8—N1—C7	−14.2 (7)
C4—C5—C6—C1	−0.9 (10)	C6—C7—N1—C11	56.7 (7)
C4—C5—C6—C7	−178.1 (6)	S1—C7—N1—C11	176.0 (4)
C1—C6—C7—N1	42.7 (8)	C6—C7—N1—C8	−134.4 (6)
C5—C6—C7—N1	−140.2 (6)	S1—C7—N1—C8	−15.1 (6)
C1—C6—C7—S1	−72.7 (7)	O3—C11—O4—C12	−0.4 (10)
C5—C6—C7—S1	104.3 (6)	N1—C11—O4—C12	179.3 (6)
N1—C8—C9—S1	37.6 (6)	C13—C12—O4—C11	−67.2 (8)
C10—C8—C9—S1	−88.4 (6)	C14—C12—O4—C11	58.1 (10)
N1—C8—C10—O1	−174.4 (6)	C15—C12—O4—C11	175.3 (6)
C9—C8—C10—O1	−53.8 (9)	C8—C9—S1—C7	−40.6 (5)
N1—C8—C10—O2	3.4 (8)	N1—C7—S1—C9	31.7 (4)
C9—C8—C10—O2	124.0 (6)	C6—C7—S1—C9	156.5 (5)

### Hydrogen-bond geometry (Å, °)

**Table:**

*D*—H···*A*	*D*—H	H···*A*	*D*···*A*	*D*—H···*A*
O2—H2A···O3^i^	0.82	1.83	2.638 (6)	167

Symmetry codes: (i) *x*+1, *y*, *z*.
