# 3-(*tert*-But­oxy­carbon­yl)-2-(4-chloro­phen­yl)-1,3-thia­zolidine-4-carb­oxy­lic acid. Corrigendum

**DOI:** 10.1107/S1600536810048774

**Published:** 2010-12-18

**Authors:** Zhong-Cheng Song, Hai-Liang Zhu, Shu-Min Ding

**Affiliations:** a State Key Laboratory of Pharmaceutical Biotechnology, School of Life Sciences, Nanjing University, Nanjing 210093, People’s Republic of China; b School of Bioengineering, Zhejiang Chinese Medical University, Hangzhou 310053, People’s Republic of China; c School of Chemical Engineering, Changzhou University, Changzhou 213164, People’s Republic of China

## Abstract

The author list in the paper by Ding [*Acta Cryst.* (2010), E**66**, o2633] is corrected.

## Abstract 2

Corrigendum to *Acta Cryst.* (2010), E**66**, o2633.

In the paper by Ding (2010) ▶, the author list is incomplete. The full list of authors is given above.
